# The risk of developing second primary malignancies among colorectal cancer patients

**DOI:** 10.18632/aging.204250

**Published:** 2022-08-26

**Authors:** Songtao Du, Yayun Li, Huiyan Sun, Guangtong Deng, Siyuan Tang, Furong Zeng, Bomiao Zhang, Binbin Cui

**Affiliations:** 1 Department of Colorectal Surgical Oncology, The Tumor Hospital of Harbin Medical University, Harbin 150001, China; 2 Department of Dermatology, Xiangya Hospital, Central South University, Changsha, Hunan 410008, China; 3 Department of Oncology, Xiangya Hospital, Central South University, Changsha, Hunan 410008, China; 4 National Engineering Research Center of Personalized Diagnostic and Therapeutic Technology, Changsha, Hunan 410008, China; 5 Department of Gastroenterology, Xiangya Hospital, Central South University, Changsha, Hunan 410008, China

**Keywords:** colorectal cancer, second primary malignancies, standardized incidence rates, lag period

## Abstract

Background: The increasing number of young colorectal cancer (CRC) survivors has led to ongoing concerns about the risk of secondary primary malignancies (SPMs). Here, we intended to comprehensively explore the pooled standardized incidence rates (SIRs) for total and site-specific SPMs in CRC survivors with different restriction to lag period.

Methods: Pubmed, Embase, Cochrane Library, and Web of science databases were searched to identify any studies reporting the SIRs of SPM following CRC until August 2021. Total and site-specific SIRs with different restriction to lag period were pooled using fixed/random effect models.

Results: A total of 42 full-text publications with more than 1, 524, 236 CRC survivors and 166, 210 SPM patients were included in the meta-analysis. Pooled data showed an increased SIRs for all SPMs in CRC survivors with different restriction to lag period (no restriction to lag period, SIR = 1.15, 95% CI = [1.08–1.23]; 1-year lag, 1.16 [1.10–1.23]; 5-year lag, 1.18 [1.09–1.28]; 10-year lag, 1.24 [1.11–1.39]). The conclusions were consistent for neoplasms of colorectum, corpus uteri, and small intestine with different restriction to lag period. However, limited evidence was presented for associations between CRC survivors and SPM for prostate, breast (female), ovarian, stomach, urinary bladder, kidney, thyroid, bone and soft tissue.

Conclusion: CRC survivors are associated with an increased risk of SPMs, especially neoplasms of colorectum, corpus uteri, and small intestine. Further studies should explore the risks for these neoplasms in CRC survivors, thus providing the reference for future follow-up care.

## INTRODUCTION

Colorectal cancer (CRC) is the third most common cancer, with an estimated 1.9 million new cases globally in 2020, accounting for 10.2% of all new cases and 935,000 related deaths [1, 2]. The combination of curative resection and adjuvant chemotherapy has become the standard therapeutic method and has achieved a significant improvement in the overall prognosis of colorectal cancer [3–5]. However, the increasing number of young colorectal cancer (CRC) survivors brought some worries about the risk of second primary malignancies (SPM) [6] which is detrimental to the prognosis of CRC survivors.

Increasing studies have served to define the risk of SPM in CRC survivors. For example, Yang et al., [7] reported that CRC survivors were at increased risk of total SPMs and second primary CRC, while Tanaka et al., [8] demonstrated that the SIRs of total SPMs and CRC cancer were not statistically significant in CRC survivors. Moreover, Ringland et al., [9] noted that the risk of second primary CRC was higher than in the general population in analysis with no restriction for latency time, while the risk was the same when restricted to studies with a lag time of 5 or 10 years. These findings suggested that it still needs to clarify the pooled SIRs for total and site-specific SPM in CRC survivors, especially with different restriction to lag period.

Here, we sought to characterize the pooled SIRs for total and site-specific SPM with restriction for different lag time in CRC survivors. Our finding will provide the reference for further follow-up care in CRC survivors.

## METHODS

This systematic review was conducted following the PRISMA (Preferred Reporting Items for Systematic Reviews and Meta-Analyses) guideline, and the systematic review protocol was registered with the International Prospective Register of Systematic Reviews (PROSPERO, http://www.crd.york.ac.uk/prospero/) on August 2021 (CRD42021276185).

### Literature search

A comprehensive systematic literature search was carried out using Pubmed, Embase, and Cochrane library and Web of Science databases to retrieve any studies that investigated the SIRs of SPM in CRC survivors from inception to August 2021. The detailed search strategies are in Supplementary Table 1. References of eligible articles were also assessed for relevant studies.

### Inclusion and exclusion criteria

For the purpose of our meta-analysis, all the studies were screened based on the following inclusion criteria: (1) CRC survivors; (2) second primary malignancies (subsequent or metachronous, not synchronous); all cancers reported subsequent to CRC were defined as second primary malignancies (subsequent or metachronous), and cancers diagnosed before the diagnosis of CRC or on the same day were regarded as synchronous malignancies. (3) Accessible SIR and its 95% confidence interval (CI). Exclusion criteria were as follows: (1) non-CRC patients; (2) studies with smaller sample size from the same authors or hospitals; and (3) patients or studies did not fulfill the inclusion criteria.

### Data extraction and quality assessment

Three reviewers (SD, FZ, and HS) summarized the eligible studies and resolved the divisions of opinion by consensus assessment. Study characteristics (first author, publication year, study population, region, gender, and mean age) and relevant data of patient characteristics (site of the second primary tumor, mean age, sex, follow-up period, SIR, 95% confidence interval, sample size, number of incidents, observed and expected patients’ number, latency time) were extracted. When SIR and its 95% CI were not reported, they were calculated based on Poisson distribution using the observed and expected incidents. Standard to identify the possible bias risk, or research rationality, in the individual studies was assessed using the Newcastle-Ottawa Scales. Studies with stars more than six were regarded as high-quality.

### Statistical analysis

STATA 16.0 (Stata Corporation, College Station, TX, USA) software was used for all the analyses. The potential heterogeneity was explored by using the Cochran’s *Q* test and I^2^ test statistics. The *p*-value < 0.1 or I^2^ > 50% indicates significant heterogeneity. The random-effect model was preferentially performed for all the analyses because of the inherent clinical heterogeneity among studies. Fixed-effect model was used to evaluate the consistency of the conclusions. Subgroup analyses stratified by sites of SPM and restriction on different lag time were performed to assess the stability of the conclusions. *P*-value less than 0.05 was considered statistically significant.

### Availability of data and materials

The datasets supporting the conclusions of this article are accessible upon reasonable requests from the corresponding authors.

## RESULTS

### Literature search and studies characteristics

**Figure 1:**
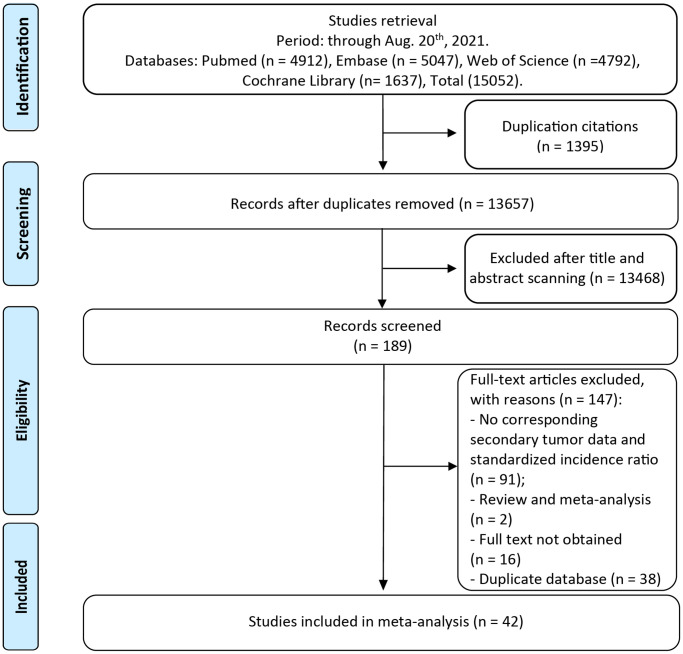
The flow chart of studies with corresponding exclusive reasons.

A total of 15,052 unique publications were retrieved by our literature search. After titles and abstracts screening, 189 articles remained for additional full-text examination (Figure 1). It is worth mentioning that 38 independent studies were eligible based on our selection criteria derived from United States Surveillance, Epidemiology, and End Results (SEER) cohort, while the most representative studies covering the broadest range of years with the largest population was kept for our analysis to avoid duplication. The full-text reviews of these articles were carefully completed and the corresponding reasons are listed in Figure 1. Finally, 42 full publications (ranged from 1969 to 2021) met our eligibility criteria, with more than 1, 524, 236 CRC survivors and 166,210 SPM patients [7–48].

The main characteristics of the eligible studies are shown in Table 1. Of the 42 publications, the principal meta-analysis on the overall risk for SPMs in CRC survivors was based on 26 studies. Other 16 articles reporting the data of a single tumor contributed to the site-specific SIRs. Thirty-four original investigations were based on regional populations, and other eight studies were conducted from different hospitals or institutions. The mean period of follow-up, reported in 28 studies, ranged from 1.5 to 16.8 years. The mean latency period (the time between diagnosis of CRC and the SPMs) ranged from 1.5 to 6.0 years. Newcastle-Ottawa Scale (NOS) was performed to assess the methodological quality of eligible studies. All studies were identified as high-quality with stars above six (Supplementary Table 2).

**Table 1:** Characteristics of included studies in meta-analysis of secondary malignancies among colorectal cancer survivals.

**Study (reference)**	**Publish Date**	**Registry (study interval)**	**Country**	**Mean age at diagnosis, (Range or SD) years**	**Male: female ratio**	**Latency,mean or SD (years)**	**Mean Follow-up (years)**	**Study Size**	**No. of Second Malignancies**
Schottenfeld [10]	1969	Memorial Sloan Kettering Cancer Center (1949–1962)	USA	–	–	–	–	4771	190
Teppo [11]	1985	Finnish Cancer Registry (1953–1979)	Finland	–	0.62	–	–	21122	329
Enblad [12]	1989	National Board of Health and Welfare the Swedish Cancer Registry (1960–1981)	Sweden	–	1.07	–	–	61769	3845
Tanaka [8]	1991	Osaka Cancer Registry (1966–1986)	Japan	60.1	1.15	–	3.68	14235	416
Levi [13]	1993	Vaud Cancer Registry (1974–1989)	Switzerland	–	1.08	–	–	–	153
Buiatti [14]	1997	Tuscany Tumour Registry (RIT), Ragusa Cancer Registry (RTR), Cancer Registry of Romagna (RTR) (1981–1989)	Italy	68.6	–	–	2.7	5238	163
McCredie [15]	1997	New South Wales Central Cancer Registry (1972–1991)	Australia	66.5	1.08	–	3.79	42509	2098
Malmer [16]	2000	The nation-wide Swedish Cancer Registry (1958–1994)	Sweden	72	–	–	–	156872	224
Evans [17]	2001	The Thames Cancer Registry (1961–1995)	England	–	0.93	–	–	127281	4317
Dong [18]	2001	The Swedish Family Cancer Database (1958–1996)	Sweden	67.6	1.14	–	2.68	67899	6197
Hemmiki [19]	2001	The Swedish Family Cancer Database (1958–1996)	Sweden	65.55	1.17	–	–	68084	5731
Green [42]	2002	U.S. national Cancer Institute (1989–1993)	USA	63.9 ± 11 (15–87)	1.22	1.5 (0.3–5.8)	1.5 (0.3–5.8)	3179	42
Moot [20]	2002	Victorian Cancer Registry (1982–1993)	Australia	66.2	–	–	7.2	13794	279
Heard [21]	2005	South Australian Cancer Registry (1977–2001)	Australia	–	–	–	–	–	1472
Bouvier [22]	2008	A population-based cancer registry in Burgundy (1976–2002)	France	71.1 (21.0–99.6)	1.24	3.6 (0.5–22.5)	–	10801	216
Cluze [23]	2009	Cancer Registry of Ise `re, France (1989–1997)	France	69.2	1.08	–	2.9	4944	224
Noura [24]	2009	Osaka Cancer Registry (1991–1996)	Japan	61 (33–89)	1.71	2.5	6.8	301	40
Ringland [9]	2010	NSW Central Cancer Registry (1987–1996)	Australia	–	1.22	Median 3.7	5.1 (1.4–10.7)	29471	660
Youlden [25]	2011	Queensland Cancer Registry (QCR) (1982–2001)	Australia	–	1.18	5.5 (1.3–10.2)	5–25	27814	3046
Raj [26]	2011	California Cancer Registry (1990–2005)	USA	64.7	1.19	2.67	6	104257	1443
Tabuchi [27]	2012	Osaka Cancer Registry (1985–2004)	Japan	–	–	–	3.9	–	2470
Dasgupta [28]	2012	Queensland, Australia 1996–2005	Australia	64	1.36	3.1	4.2 (2.2–7.3)	15755	1615
Kok [29]	2012	Netherlands Cancer Registry (1989–2008)	Netherlands	–	–	–	–	–	–
Mulder [30]	2012	Rotterdam Cancer registry in the Netherlands (1995–2006)	Netherlands	70.0 (62–77)	1.02	–	3.9	10283	135
Levi [31]	2012	Vaud Cancer Registry (1974–2008)	Switzerland	–	1.15	–	4.7	9389	136
Tabuchi [32]	2013	Osaka Medical Center for Cancer and Cardiovascular Diseases (1985–2004)	Japan	–	–	–	5.2	2155	204
Utada [33]	2014	Nagasaki Prefecture Cancer Registry (1985–2007)	Japan	–	–	–	4.3	–	2997
Jégu [34]	2014	K2 France nationwide study (1984–2004)	France	64.2	–	–	0.16–18	–	2929
Coyte [35]	2014	Scottish Cancer Registry (2000–2004)	Scotland	69.9 ± 11.7	1.13	–	–	7225	324
Lee [36]	2015	Taiwan’s national Health Insurance (1996–2011)	China	67 (56–75)	1.29	4.7 (2.7–7.5)	4.03 (2.14–7.49)	98876	4259
Liang [37]	2015	Taiwan Cancer Registry (TCR) (1995–2005)	China	66	1.32	–	4.4	65 648	3810
Kato [38]	2016	Saitama Medical Center (2007–2011)	Japan	67.4 ± 11.2	1.73	1.5 (3–61)	3.69 (1.6)	1005	126
Preyer [39]	2017	Tyrol and Vorarlberg Cancer Registries (1988–2005)	Austria	–	–	–	5.7 (1.4–10.3)	7138	614
Yang [7]	2017	SEER (1973–2012)	USA	68 (14–102)	1.36	–	6.97	288390	33047
Kim [40]	2017	Republic of Korea national Health Insurance System database (2007–2012)	Korea	–	–	–	5.78	85455	2005
Chung [41]	2017	Severance Hospital (2001–2009)	Korea	61.0 (45.0–74.0)	1.62	0.3 (0.8–10)	3.3 (0–30.9)	4822	
Guan [43]	2015	SEER (1992–2012)	USA	–	1.42	–	–	240584	27731
He [44]	2018	SEER (1973–2013)	USA	–	–	–	–		50679
Bright [45]	2019	The Teenage and Young Adult Cancer Survivor Study England and Wales (1971–2006)	England	–	1.03	–	16.8	5805	537
Ahn [46]	2019	Support for Serious Illness (SSI) program Korean NHI claims database (2005–2015)	Korean	65.56 ± 10.51	1.58	3.08 (1.87–3.44)	–	251482	498
Feller [47]	2020	Swiss cantonal cancer registries (1981–2009)	Switzerland	–	–	6.0 (2.1–10.9)	More than 5	35949	4441
Tanaka [48]	2021	Japan Clinical Oncology Group JCOG0205, JCOG0212 and JCOG0404	Japan	62 (23–75)	1.38	–	6.0 (5.0–7.2)	2824	240

Abbreviation: SEER: Surveillance, Epidemiology, and End Results.

### Site-specific prevalence of SPM in CRC survivors

On unadjusted analysis with no restriction to lag period, the pooled site-specific prevalence is 1.506% for prostate cancer, 1.202% for colorectal cancer, 1.060% for breast cancer, with less than 1.00% other malignancies (Supplementary Figure 1).

### Overall and site-specific SIRs of SPM in CRC survivors

**Figure 2:**
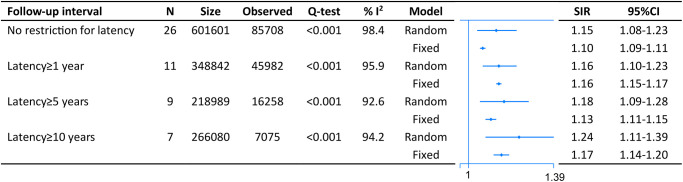
The pooled standardized incidence rates (SIR) for overall second primary malignancies (SPM) in CRC survivors with different restriction to lag time.

On pooled analysis with data derived from 26 studies comprising 601,601 CRC survivors and 85,708 SPM patients, we found an increased risk of second malignancies with no restriction to lag period (SIR = 1.15, 95% CI = [1.08–1.23]). The result was similar when we restricted analysis with lag one (SIR = 1.16, 95% CI = [1.10–1.23]), five (SIR = 1.18, 95% CI = [1.09–1.28]) or ten (SIR = 1.24, 95% CI = [1.11–1.39]) years (Figure 2).

**Figure 3:**
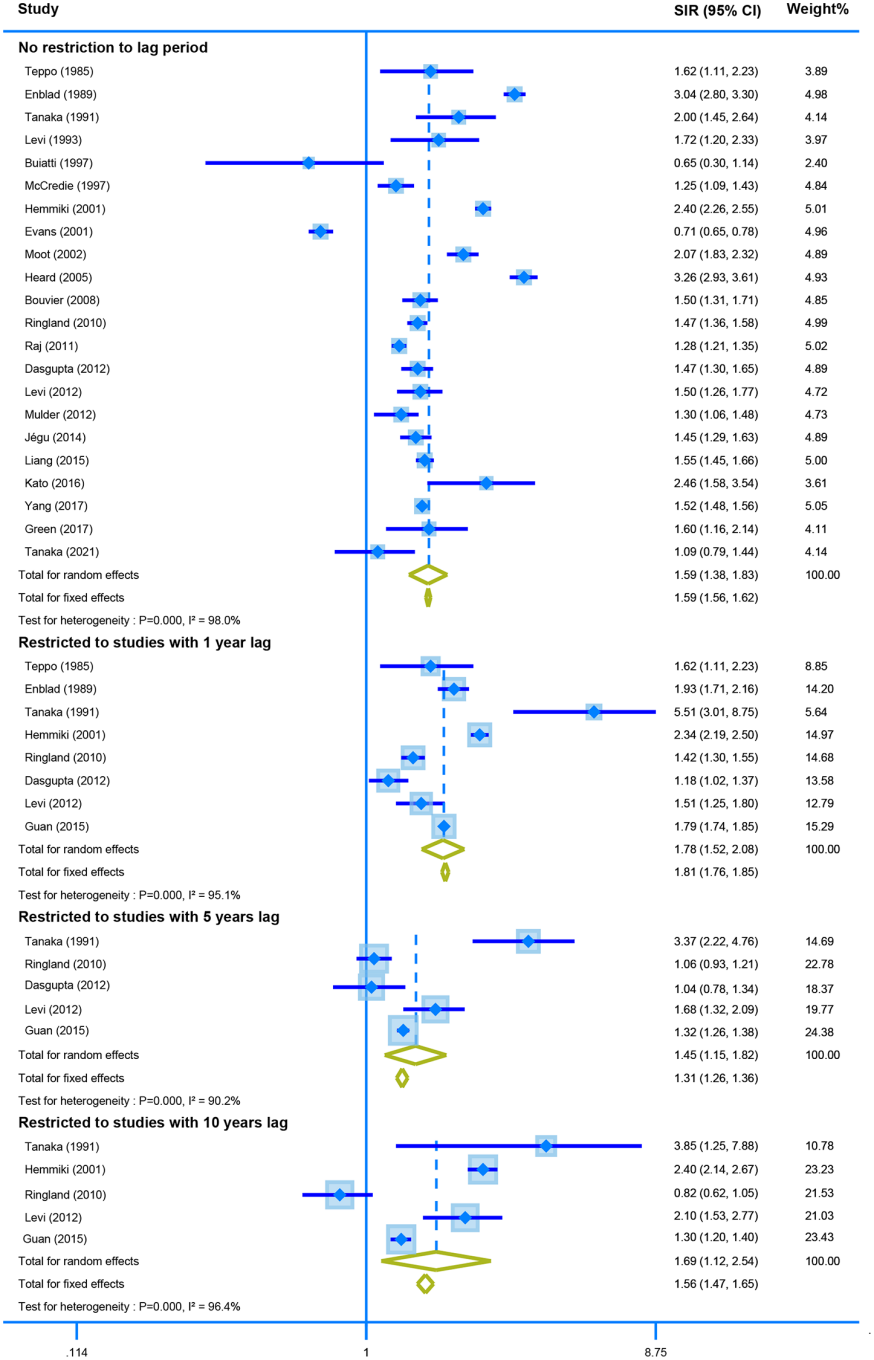
The pooled SIR for second colorectal cancer in CRC survivors with different restriction to lag time.

Twenty-two studies with no restriction for latency time reported the association between CRC survivors and second primary CRC. The random-effect model showed that CRC survivors had a higher risk of developing second CRC (SIR = 1.59, 95% CI = [1.38–1.83]). Similar results were obtained when the analysis was restricted by one, five-, and ten-years lag (one-year lag, SIR = 1.78, 95% CI = [1.52–2.08]; five-years lag, SIR = 1.45, 95% CI = [1.15–1.82]; and ten-years lag, SIR = 1.69, 95% CI = [1.12–2.54]) (Figure 3).

**Figure 4:**
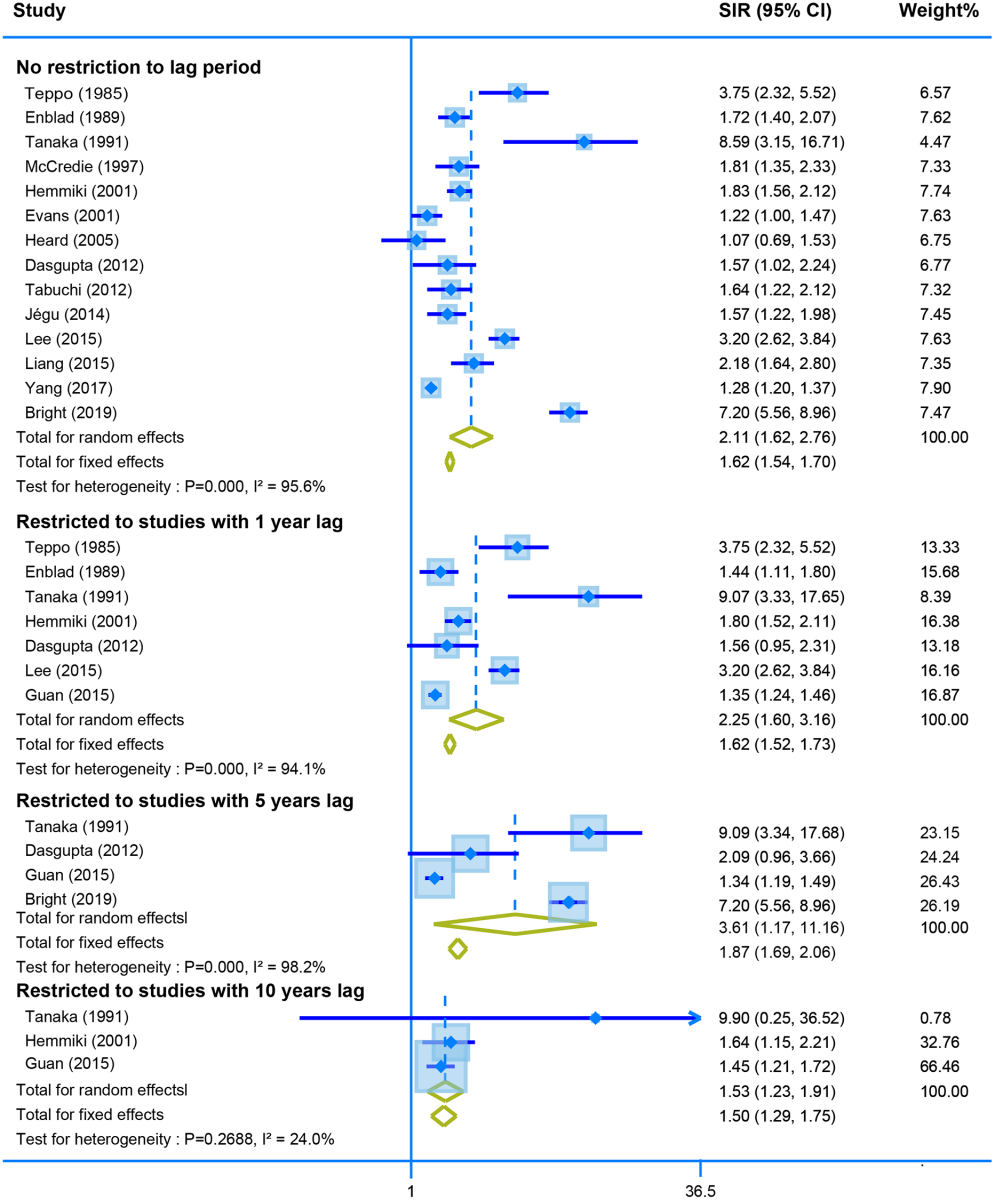
The pooled SIR for second neoplasm of corpus uteri in CRC survivors with different restriction to lag time.

Fourteen studies reported the SIR of the second neoplasm of uterine corpus in CRC survivors with no restriction for latency time. The pooled data showed a higher risk of developing the malignant neoplasm of corpus uteri using the random model (SIR = 2.11, 95% CI = [1.62–2.76]). Moreover, the SIR was still similar when we calculated the derived data stratified by different lag time (one-year lag, SIR = 2.25, 95% CI = [1.60–3.16]; five-years lag, SIR = 3.61, 95% CI = [1.17–11.16]; and ten--years lag, SIR = 1.53, 95% CI = [1.23–1.91]) (Figure 4).

**Figure 5:**
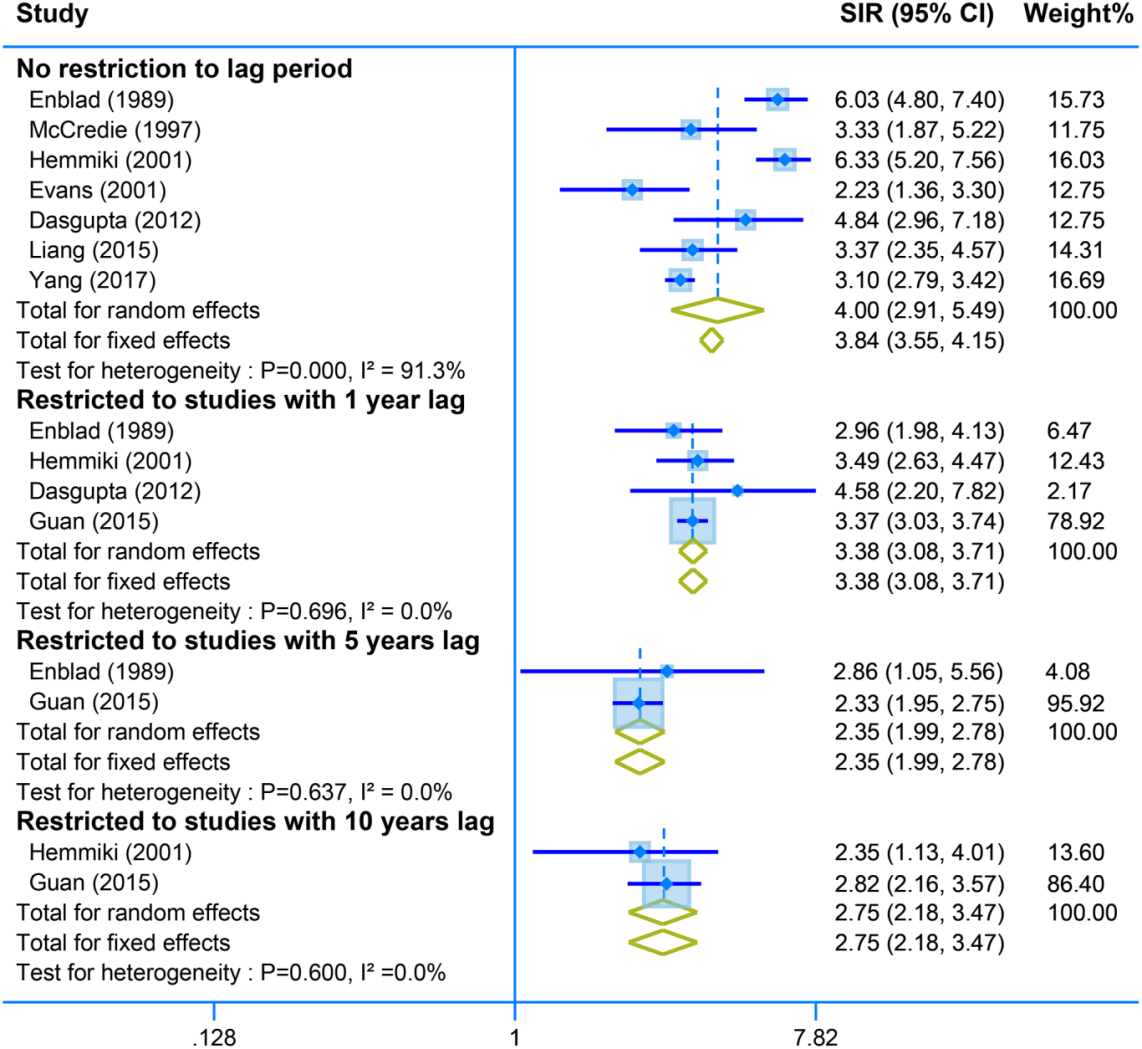
The pooled SIR for second neoplasm of small intestine in CRC survivors with different restriction to lag time.

Pooled analysis of seven studies with no restriction to lag time showed a positive association between second malignant of the small intestine and CRC survivors (SIR = 4.00, 95% CI = [2.91–5.49]. Restriction of the analysis with different years lag presents stable results of high risk of small intestine tumor (one-year lag, SIR = 3.38, 95% CI = [3.08–3.71]; SIR = 2.35, 95% CI = [1.99–2.78]; and SIR = 2.75, 95% CI = [2.18–3.47]) (Figure 5).

We also found that CRC survivors are more likely to develop second neoplasms of prostate, breast (female), ovarian, stomach, urinary bladder, kidney, thyroid, bone and soft tissue (Supplementary Figure 1). However, the results of the above tumors failed to be consistent with the analysis stratified by different lag time (Supplementary Figures 2–9). Moreover, there were no increasing association between CRC survivors and neoplasms of stomach, oral, lymphoma, pancreas, leukemia, brain, cervix, esophagus, larynx and gall bladder (Supplementary Figure 1).

## DISCUSSION

An increasing number of young CRC survivors are confronted with the threat of developing SPM [6]. Appropriate risk assessment for SPMs has substantial therapeutic implications for long-term patient surveillance and the reduction of morbidity. In this study, based on the 42 publications from comprehensive population databases worldwide, we carried out a meta-analysis to explore the risk of overall SPMs in CRC survivors. Our results demonstrated that CRC survivors were at increased risk of developing second tumors, especially neoplasms of colorectum, corpus uteri, and small intestine.

To our knowledge, this is the first meta-analysis to comprehensively evaluate the risk of SPMs in CRC survivors. Previously, Keegan et al., demonstrated that adolescents and young adults with secondary neoplasms were more likely to experience worse survival compared with adolescents and young adults with the same primary neoplasms [49]. This finding highlights the importance of active surveillance for CRC patients. However, studies conducted by Levi et al., and Youlden et al., mainly focused on overall SPMs risk, but ignored site-specific SIRs [13, 25]. The site-specific SIRs could precisely facilitate the management of disease surveillance in CRC survivors. In this point, our study is of great significance in the field.

Our results confirmed the positive association between SPMs and CRC history [50, 51], and the reasons may be as follows, (1) genetic predisposition and environmental exposures; [9, 30, 52, 53] (2) adjuvant therapy, such as radiotherapy [54] and chemotherapy [55]. Green et al., demonstrated that CRC survivors receiving adjuvant chemotherapy had the high incidence of second primary colorectal cancer. Moreover, direct radiation, radiation scatter or radiation induced genetic alterations with direct exposure might contribute to carcinogenesis due to increased reactive oxygen species and changes of gene expression [56]. Further studies are needed to elucidate underlying mechanisms for the association between CRC survivors and its SPMs.

Inevitably, there are several limitations related to this meta-analysis. First, significant heterogeneity existed among the analysis, while different effect models and subgroup analyses showed a unified result, which further confirmed the conclusion. Second, some SIRs and their 95% CI were estimated based on Poisson distribution using the observed and expected cases, which could cause some bias of results. Finally, we were unable to adjust for any heterogeneity in treatment between studies or evaluate risks by different treatment modalities, as treatment data was not available for the majority of studies.

## CONCLUSION

In summary, for the first time, we reported that CRC survivors are associated with an increased risk of SPMs, especially neoplasms of colorectum, corpus uteri, and small intestine. Further studies should explore the risks for these neoplasms in CRC survivors, thus providing the reference for future follow-up care.

## Supplementary Materials

Supplementary Figures

Supplementary Tables
