# Supplementary material for: The risk of developing second primary malignancies among colorectal cancer patients
Source: Aging (Albany NY). 2022 Aug 26;14(16):6756–79. doi: 10.18632/aging.204250 (PMC9467398; doi:10.18632/aging.204250)
Supplement: Supplementary Figures [file aging-14-204250-s001.pdf]

SUPPLEMENTARY FIGURES

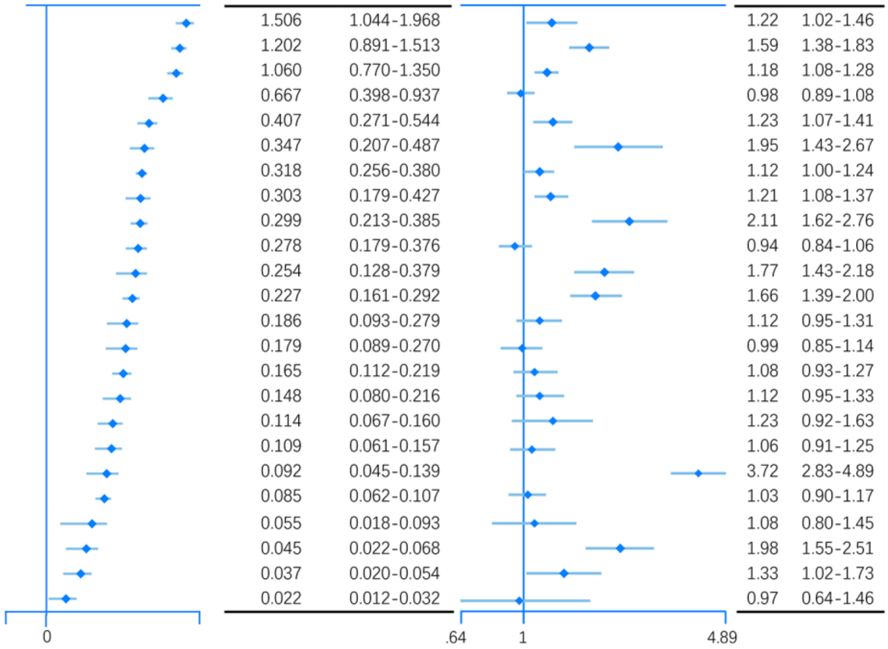

Tumor	N	Size	Observed	%Prevalence	95%CI	SIR	95%CI
Prostate	18	769457	13009	1.506	1.044-1.968	1.22	1.02-1.46
Colorectal	22	796736	12295	1.202	0.891-1.513	1.59	1.38-1.83
Breast (female)	16	756659	5889	1.060	0.770-1.350	1.18	1.08-1.28
Lung & bronchus	17	754136	8914	0.667	0.398-0.937	0.98	0.89-1.08
Skin	8	382341	969	0.407	0.271-0.544	1.23	1.07-1.41
Ovary	11	669678	1033	0.347	0.207-0.487	1.95	1.43-2.67
Stomach	17	752189	3716	0.318	0.256-0.380	1.12	1.00-1.24
Bladder	14	749064	3607	0.303	0.179-0.427	1.21	1.08-1.37
Corpus uteri	14	743826	1730	0.299	0.213-0.385	2.11	1.62-2.76
Liver	9	658936	1889	0.278	0.179-0.376	0.94	0.84-1.06
Vagina & genital female	7	593207	415	0.254	0.128-0.379	1.77	1.43-2.18
Kidney	12	739600	1720	0.227	0.161-0.292	1.66	1.39-2.00
Oral	6	341475	833	0.186	0.093-0.279	1.12	0.95-1.31
Lymphoma	10	623828	1754	0.179	0.089-0.270	0.99	0.85-1.14
Pancreas	15	979008	2396	0.165	0.112-0.219	1.08	0.93-1.27
Leukemia	9	623828	1310	0.148	0.080-0.216	1.12	0.95-1.33
Brain	7	712972	723	0.114	0.067-0.160	1.23	0.92-1.63
Cervix	8	722266	380	0.109	0.061-0.157	1.06	0.91-1.25
Small intestine	7	603788	655	0.092	0.045-0.139	3.72	2.83-4.89
Esophagus	10	697485	827	0.085	0.062-0.107	1.03	0.90-1.17
Larynx	4	392668	324	0.055	0.018-0.093	1.08	0.80-1.45
Thyroid	7	412754	229	0.045	0.022-0.068	1.98	1.55-2.51
Bone & softtissue	5	582631	257	0.037	0.020-0.054	1.33	1.02-1.73
Gallbladder	5	451028	163	0.022	0.012-0.032	0.97	0.64-1.46

Supplementary Figure 1. Meta-analysis of site-specific SIRs in CRC survivors.

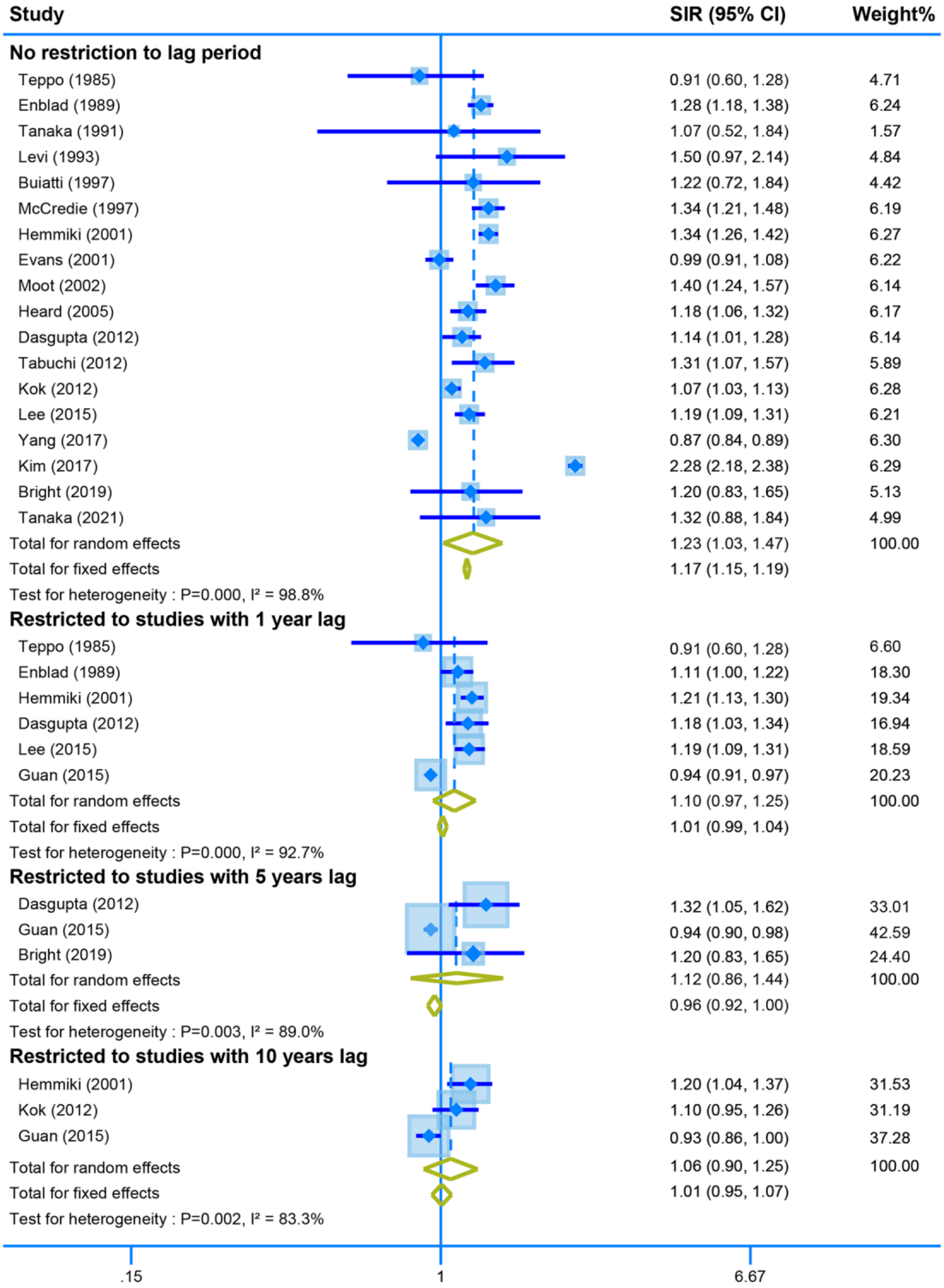

Supplementary Figure 2. The pooled SIR for second prostate cancer in CRC survivors with different restriction to lag time.

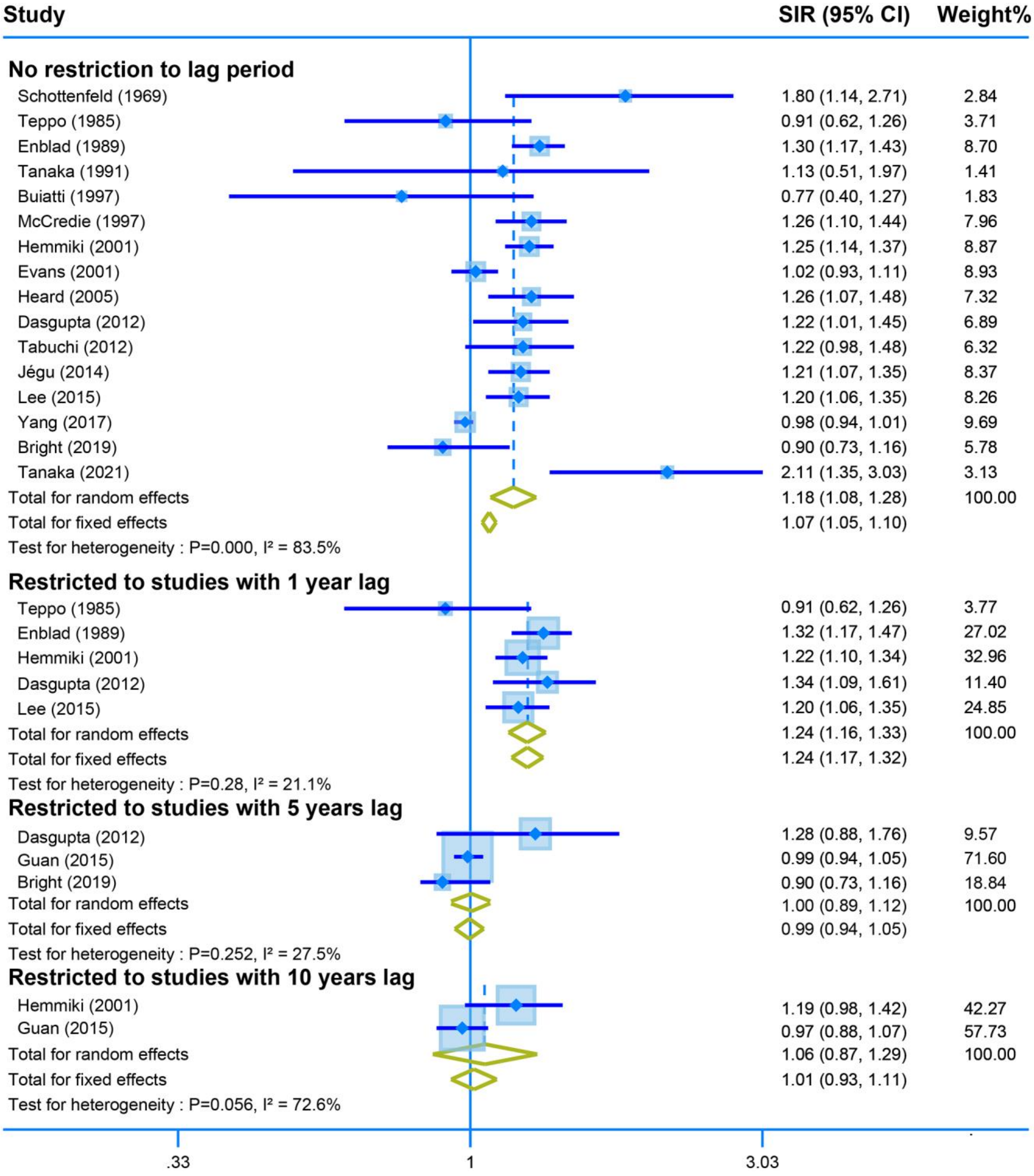

Supplementary Figure 3. The pooled SIR for second breast cancer in CRC survivors with different restriction to lag time.

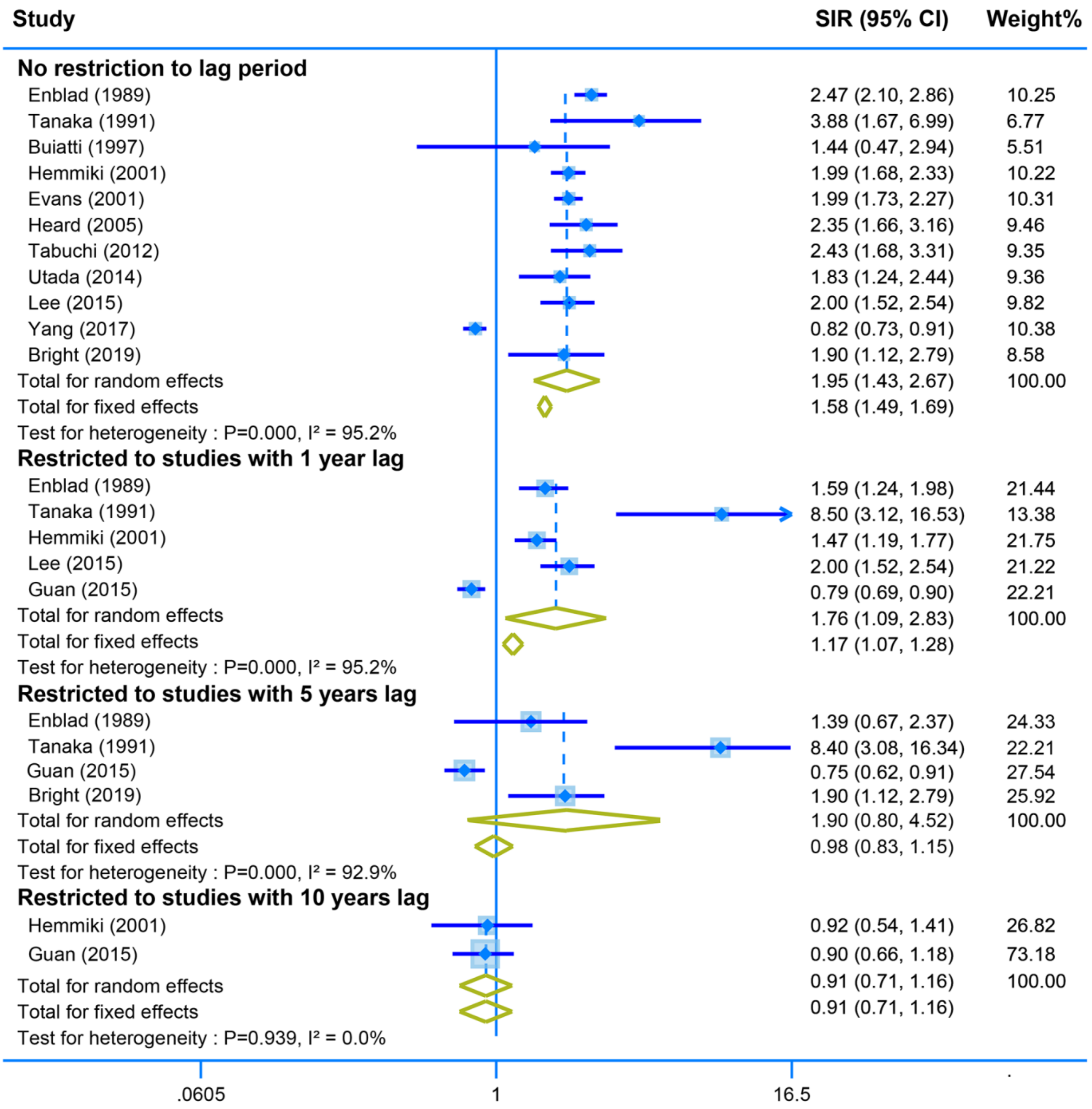

Supplementary Figure 4. The pooled SIR for second ovary cancer in CRC survivors with different restriction to lag time.

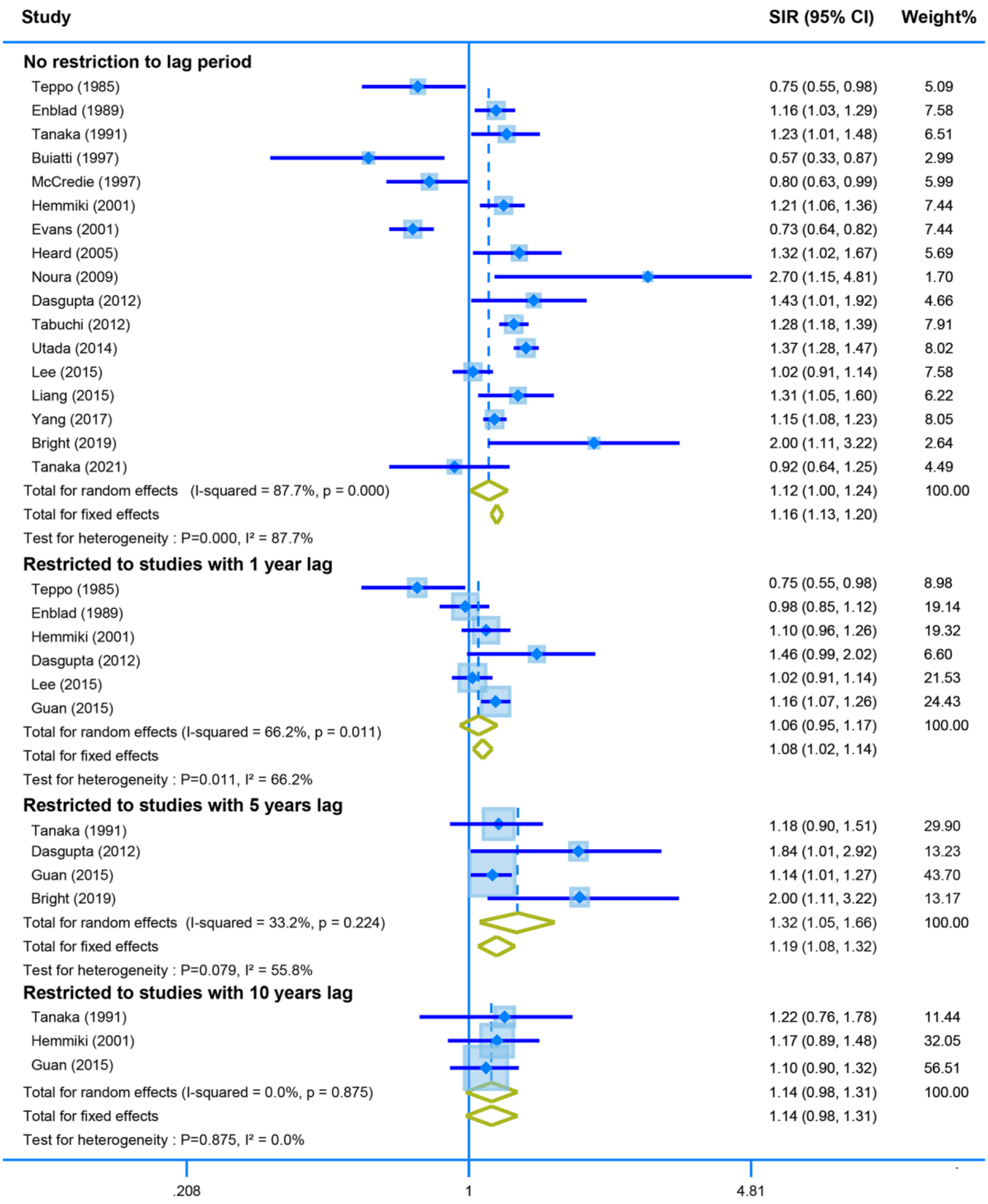

Supplementary Figure 5. The pooled SIR for second neoplasm of stomach in CRC survivors with different restriction to lag time.

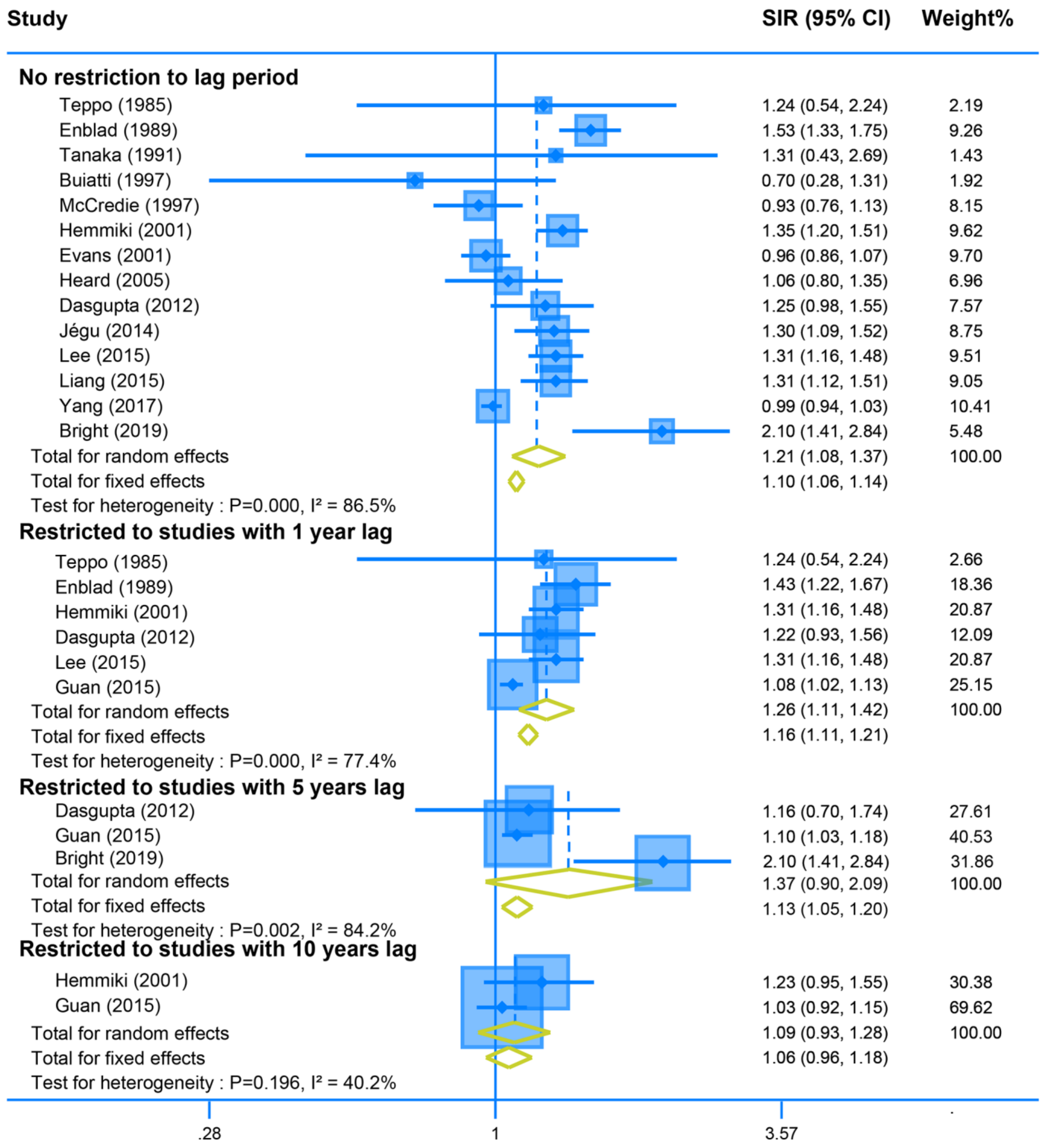

Supplementary Figure 6. The pooled SIR for second bladder cancer in CRC survivors with different restriction to lag time.

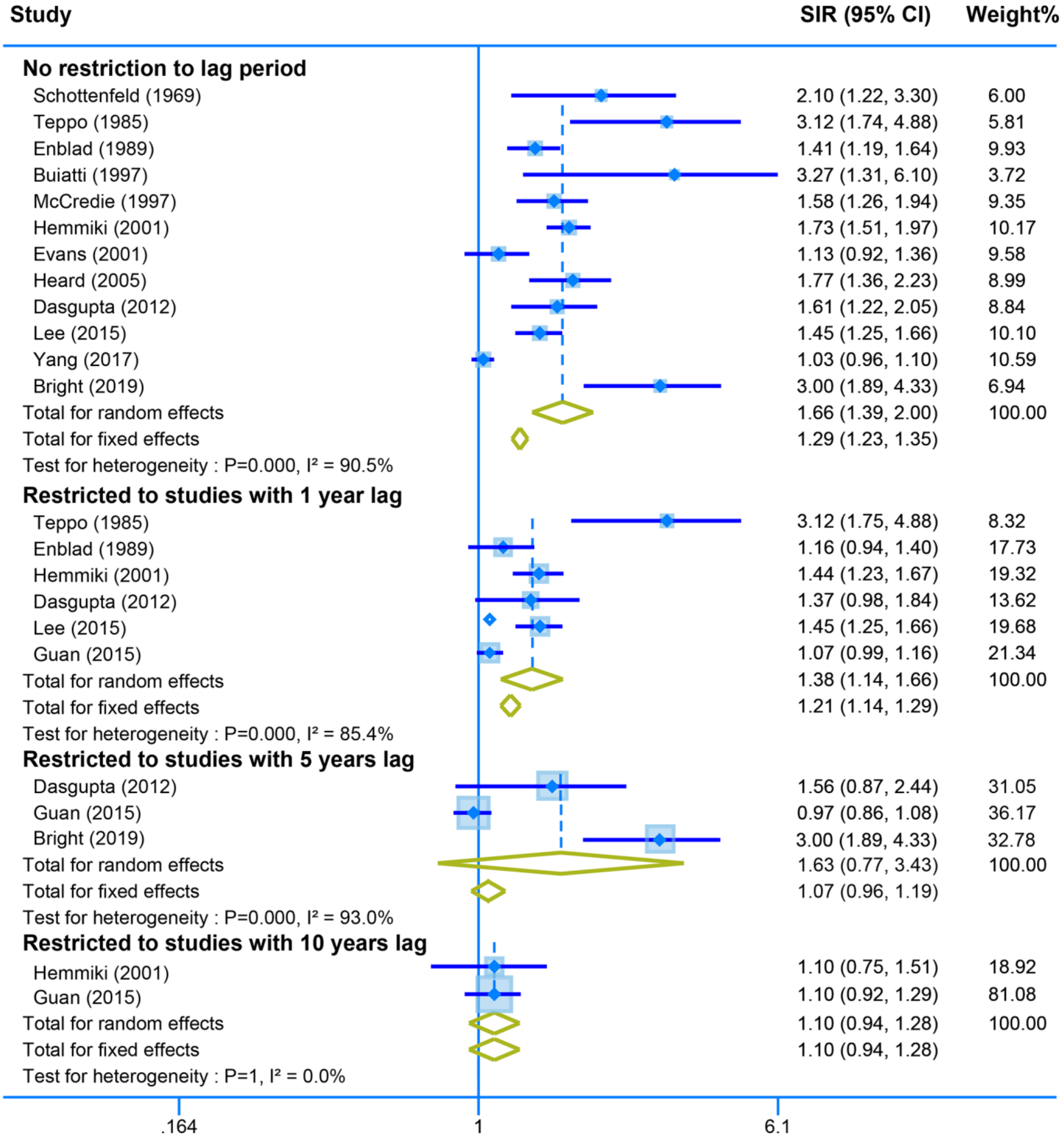

Supplementary Figure 7. The pooled SIR for second kidney cancer in CRC survivors with different restriction to lag time.

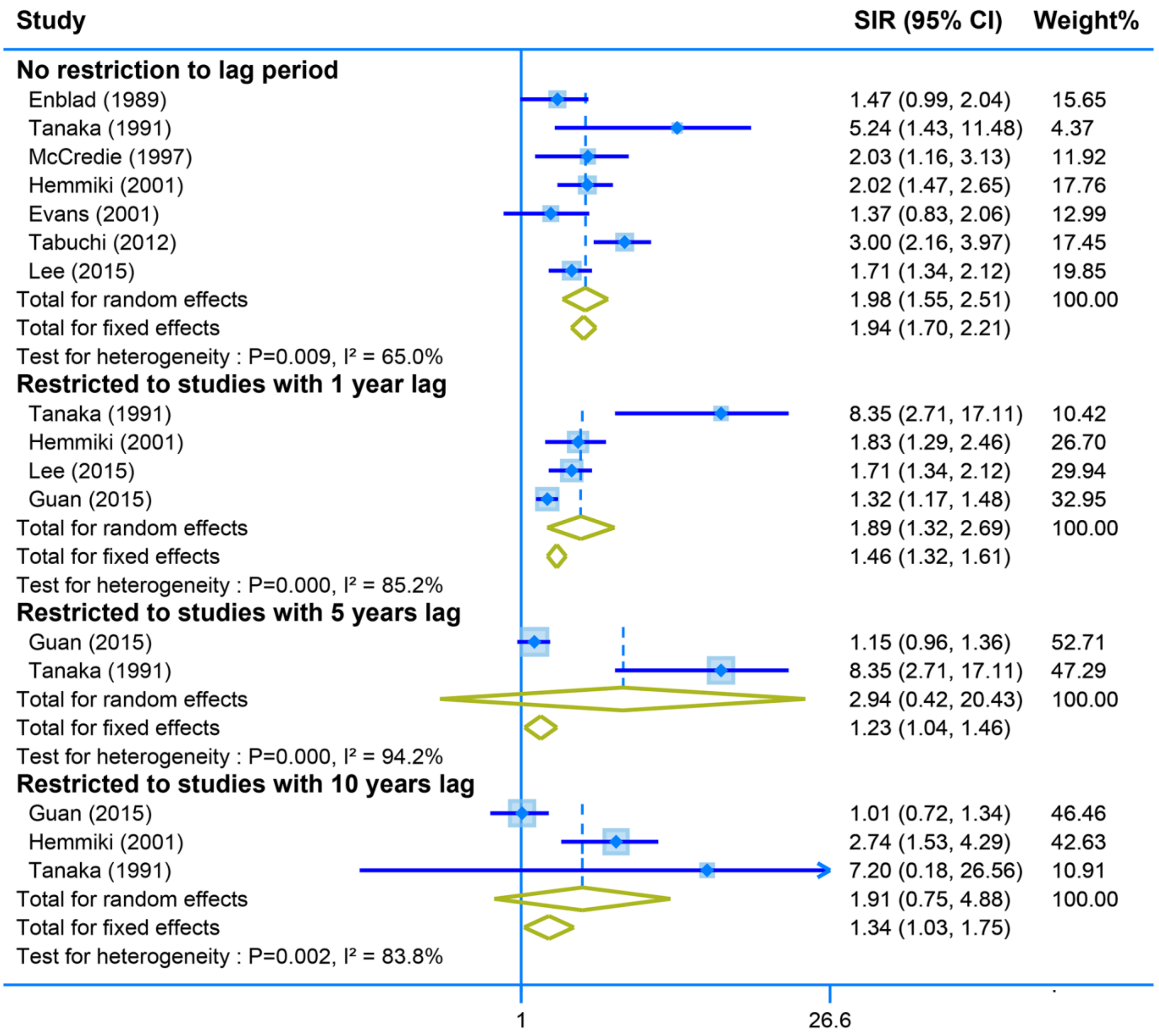

Supplementary Figure 8. The pooled SIR for second thyroid cancer in CRC survivors with different restriction to lag time.

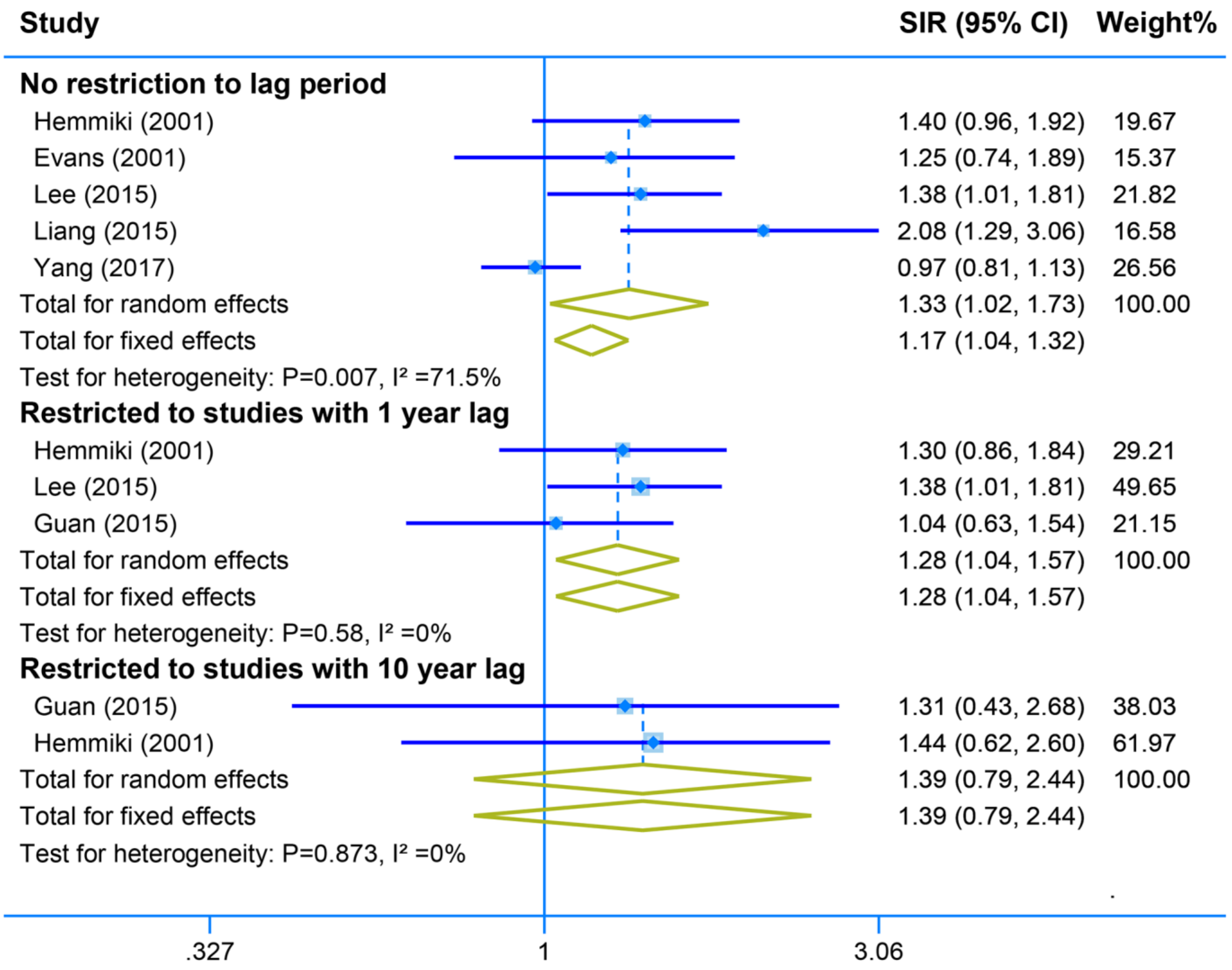

Supplementary Figure 9. The pooled SIR for second neoplasm of bone and soft tissue in CRC survivors with different restriction to lag time.
