# Supplementary material for: The risk of developing second primary malignancies among colorectal cancer patients
Source: Aging (Albany NY). 2022 Aug 26;14(16):6756–79. doi: 10.18632/aging.204250 (PMC9467398; doi:10.18632/aging.204250)
Supplement: Supplementary Tables [file aging-14-204250-s002.pdf]

SUPPLEMENTARY TABLES

Supplementary Table 1. The detailed search strategy.

Databases	Search strategy
**Pubmed**	#1 (Colorectal Neoplasms[Mesh]) OR (colorect* or colon* or rect* or anal* or anus* or intestin* or bowel*) and (carcinom* or neoplas* or adenocarcinom* or cancer* or tumor* or tumour*)
	#2 second cancer[tw] OR second tumor[tw] OR second neoplasm[tw] OR second primary[tw] OR second malignancy[tw]
	#3 risk[mesh] OR risk*[tw] OR incidence*[tw] OR prevalence*[tw] OR epidemiol*[tw]
	#4 #1 AND #2 AND #3
	#5 animals[mesh] NOT humans[mesh]
	#6 #4 NOT #5
**Embase**	#1 (exp Colorectal Neoplasms) OR (colorect* or colon* or rect* or anal* or anus* or intestin* or bowel*) and (carcinom* or neoplas* or adenocarcinom* or cancer* or tumor* or tumour*)
	#2 (second cancer OR second tumor OR second neoplasm OR second primary OR second malignancy):ti,ab,de
	#3 (risk* OR incidence* OR prevalence* OR epidemiol*):ti,ab,de
	#4 #1 AND #2 AND #3
	#5 [animals]/lim NOT [humans]/lim
	#6 #4 NOT #5
**Web of Science**	#1 (Colorectal Neoplasms/exp) OR (colorect* or colon* or rect* or anal* or anus* or intestin* or bowel*) and (carcinom* or neoplas* or adenocarcinom* or cancer* or tumor* or tumour*)
	#2 second cancer OR second tumor OR second neoplasm OR second primary OR second malignancy
	#3 (risk* OR incidence* OR prevalence* OR epidemiol*)
	#4 #1 AND #2 AND #3
**Cochrane**	#1 MeSH descriptor: [colorectal cancer] explode all tree
	#2 (Colorectal Neoplasms) OR (colorect* or colon* or rect* or anal* or anus* or intestin* or bowel*) and (carcinom* or neoplas* or adenocarcinom* or cancer* or tumor* or tumour*)
	#3 #1 OR #2
	#4 ((cancer* OR tumo* OR carcinom* OR neoplas* OR malignanc* OR squam* OR epitheliom* OR melanom* OR lymphom*) NEAR/1 (multiple OR subsequen* OR another OR further OR more OR second* OR metachron*))
	#5 risk* OR incidence* OR prevalence* OR epidemiol*
	#6 #3 AND #4 AND #5

Supplementary Table 2. Risk of quality assessment of studies included in the meta-analysis based on Newcastle-Ottawa Scale.

Study	Date	Selection*	Comparability#		Exposure/Outcome‡		Total score
		Score	Score	Notes	Score	Notes	
Tanaka	2021	★★★★	★★		★★★	–	9
Schottenfeld	1969	★★★	★★		★★	No description of follow-up time	7
Evans	2001	★★★	★★		★★	Less than 5 years of mean follow-up time	7
Tabuchi	2013	★★★	★★		★★★	–	8
Tabuchi	2012	★★★	★★		★★	Less than 5 years of mean follow-up time	7
Teppo	1985	★★★	★★		★★★	–	8
Enblad	1989	★★★	★★		★★★	–	8
Tanaka	1991	★★★	★★		★★	Less than 5 years of mean follow-up time	7
Levi	1993	★★★	★★		★★	No description of follow-up time	7
Buiatti	1997	★★★	★	No description	★★★	–	7
McCredie	1997	★★★	★★		★★	Less than 5 years of mean follow-up time	7
Dong	2001	★★★	★	No description	★★★	–	7
Hemmiki	2001	★★★	★★		★★★	–	8
Heard	2005	★★★	★★		★★	A proportion did not complete the follow-up	7
Cluze	2009	★★★	★★		★★	Less than 5 years for statistics of second tumors	7
Noura	2009	★★★	★★		★★★	–	8
Youlden	2011	★★★	★	No description	★★★	–	7
Dasgupta	2012	★★★	★★		★★	Less than 5 years of mean follow-up time	7
Utada	2014	★★★	★★		★★	Less than 5 years of mean follow-up time	7
Jégu	2014	★★★	★★		★★★	–	8
Coyte	2014	★★★	★★		★★	No description of follow-up time	7
Lee	2015	★★★	★★		★★	Less than 5 years of mean follow-up time	7
Liang	2015	★★★	★★		★★★		8
Preyer	2017	★★★	★★		★★★	–	8
Yang	2017	★★★	★★		★★★	–	8
Guan	2015	★★★	★★		★★★	–	8
He	2018	★★★	★★		★★★	–	8
Bright	2019	★★★	★★		★★★	–	8
Feller	2020	★★★	★★		★★★	–	8
Malmer	2000	★★★	★★		★★★	–	8
Moot	2002	★★★	★★		★★★	–	8
Bouvier	2008	★★★	★★		★★★	–	8
Ringland	2010	★★★	★★		★★★	–	8
Raj	2011	★★★	★★		★★★	–	8
Kok	2012	★★★	★★		★★★	–	8
Mulder	2012	★★★	★★		★★★	–	8
Levi	2012	★★★	★★		★★★	–	8
Kato	2016	★★★	★★		★★	Less than 5 years of mean follow-up time	7
Kim	2017	★★★	★★		★★★	–	8
Chung	2017	★★★	★★		★★	Less than 5 years of mean follow-up time	7
Green	2017	★★★	★★		★★	Less than 5 years of mean follow-up time	7
Ahn	2019	★★★	★★		★★	A proportion did not complete the follow-up	7

*Maximum score of 4, since most of the studies were retrospective analyses, they did not score full marks. #Maximum score of 2. ‡Maximum score of 3, even though the mean follow-up of a study was no more than five years, the data on secondary tumors of more than ten years was detailed. The study was considered as a complete follow-up.
